# Collagen VI Deficiency Impairs Tendon Fibroblasts Mechanoresponse in Ullrich Congenital Muscular Dystrophy

**DOI:** 10.3390/cells13050378

**Published:** 2024-02-22

**Authors:** Vittoria Cenni, Patrizia Sabatelli, Alberto Di Martino, Luciano Merlini, Manuela Antoniel, Stefano Squarzoni, Simona Neri, Spartaco Santi, Samuele Metti, Paolo Bonaldo, Cesare Faldini

**Affiliations:** 1 CNR-Institute of Molecular Genetics, via di Barbiano 1/10, 40136 Bologna, Italysquarzoni@area.bo.cnr.it (S.S.); spartaco.santi@cnr.it (S.S.); 2 IRCCS Istituto Ortopedico Rizzoli, 40136 Bologna, Italy; 3 1st Orthopedics and Traumatology Department, IRCCS Istituto Ortopedico Rizzoli, 40136 Bologna, Italy; albertocorrado.dimartino@ior.it (A.D.M.); cesare.faldini@ior.it (C.F.); 4 Department of Biomedical and Neuromotor Science, DIBINEM, University of Bologna, 40136 Bologna, Italy; mrllcn@unife.it; 5 Medicine and Rheumatology Unit, IRCCS Istituto Ortopedico Rizzoli, 40136 Bologna, Italy; simona.neri@ior.it; 6 Department of Molecular Medicine, University of Padova, 35122 Padova, Italy; samuele.metti@unipd.it (S.M.); paolo.bonaldo@unipd.it (P.B.)

**Keywords:** collagen VI, Ullrich congenital muscular dystrophy, COL6-related myopathies, contractures, tendon fibroblasts, mechanosignaling, primary cilium, focal adhesion

## Abstract

The pericellular matrix (PCM) is a specialized extracellular matrix that surrounds cells. Interactions with the PCM enable the cells to sense and respond to mechanical signals, triggering a proper adaptive response. Collagen VI is a component of muscle and tendon PCM. Mutations in collagen VI genes cause a distinctive group of inherited skeletal muscle diseases, and Ullrich congenital muscular dystrophy (UCMD) is the most severe form. In addition to muscle weakness, UCMD patients show structural and functional changes of the tendon PCM. In this study, we investigated whether PCM alterations due to collagen VI mutations affect the response of tendon fibroblasts to mechanical stimulation. By taking advantage of human tendon cultures obtained from unaffected donors and from UCMD patients, we analyzed the morphological and functional properties of cellular mechanosensors. We found that the length of the primary cilia of UCMD cells was longer than that of controls. Unlike controls, in UCMD cells, both cilia prevalence and length were not recovered after mechanical stimulation. Accordingly, under the same experimental conditions, the activation of the Hedgehog signaling pathway, which is related to cilia activity, was impaired in UCMD cells. Finally, UCMD tendon cells exposed to mechanical stimuli showed altered focal adhesions, as well as impaired activation of Akt, ERK1/2, p38MAPK, and mechanoresponsive genes downstream of YAP. By exploring the response to mechanical stimulation, for the first time, our findings uncover novel unreported mechanistic aspects of the physiopathology of UCMD-derived tendon fibroblasts and point at a role for collagen VI in the modulation of mechanotransduction in tendons.

## 1. Introduction

Collagen VI is a distinctive extracellular matrix (ECM) protein widely expressed in most tissues, including skeletal muscle, tendons, peripheral nerves, and skin, that forms a network of beaded filaments anchored to the cell surface [1]. The most abundant form of collagen VI is the [α1(VI)α2(VI)α3(VI)] triple-helical monomer that further assembles intracellularly into dimers and tetramers. After secretion, tetramers undergo end-to-end association, giving rise to characteristic 100 nm-spaced beaded microfibrils [2]. Depending on their association with cell receptors and ECM-binding proteins, collagen VI microfibrils may either form fibrils by parallel alignment, or web-like structures by multiple interconnections [2]. Two additional collagen VI subunits, the α5(VI) and α6(VI) chains, structurally resemble the α3(VI) chain but display a more restricted, and often alternative, distribution pattern [3].

Mutations in *COL6A1*, *COL6A2*, and *COL6A3* genes cause a group of myopathies (COL6-related myopathies, or COL6-RMs) [4], which comprise two major clinical forms, Bethlem myopathy (BM, MIM #158810) and Ullrich congenital muscular dystrophy (UCMD, MIM #254090) [5], and a phenotype in between UCMD and BM, referred to as intermediate COL6-RM, or the myosclerosis myopathy variants (MM, MIM #255600) [6].

In addition to variable muscle weakness, patients affected by COL6-RM display different degrees of axial and proximal joint contractures together with distal joint hypermobility, which worsen motor capability and deeply affect patients’ quality of life [7]. Therefore, understanding the mechanisms underlying the onset of contractures is crucial for the development of effective therapeutic strategies in COL6-RM. Increasing evidence points to tendon dysfunction as a possible cause. Tendon alterations including the disruption of the pericellular matrix (PCM) network, defective cell polarization, and structural changes of collagen fibrils, have indeed been described in tendon biopsies and tendon-derived cell cultures of BM and UCMD patients [8,9,10]. *Col6a1^−/−^* mice, a collagen VI null model [11], *Col6a3* deficient mice [12], and *Col6a* null mice [13] display reduced tendon fibril size and mechanical properties, suggesting that collagen VI plays a key role in tendon function. While such evidence highlights an involvement of the tendon structure in the pathogenesis of COL6-RM, the underlying molecular mechanism and the impact on tendon cell function have been poorly explored.

Tendons are composed of dense connective tissue arranged in a highly ordered ECM, mainly constituted by collagen fibrils, hierarchically organized to withstand tensile forces from muscles to the bone axis. Besides fibrils, the ECM is composed by relatively rare cells represented by tendon fibroblasts (TFs) or tenocytes, depending on their state of activation [14]. Fibrils mostly contain collagen types I, III, V, VI, XII, and XIV, as well as proteoglycans and glycoproteins [15]. Tenocytes, the ECM-producing cells, are distributed longitudinally among collagen fascicles and are surrounded by a PCM consisting of specialized ECM proteins, that also include collagen VI [16,17,18]. By interacting with cell membrane receptors [8] and other collagens [19], collagen VI forms a microfibrillar scaffold that envelops the cell and regulates several cell surface-mediated functions such as migration, proliferation, and fibrillogenesis. The unique structure of the PCM creates a local mechanical environment that modulates the matrix-induced deformation on tendons cells, and allows tenocytes to respond to external stimuli, transducing mechanical signals into chemical cues. Several cellular structures including cilia, focal adhesions, and the cytoskeleton interact with ECM components, allowing tendon cells to sense and convert these mechanical stimuli into biochemical signals.

The primary cilium (PC) is an antenna-like, non-motile organelle that protrudes from almost all cell types in the ECM [20]. PC shape is supported by a microtubular-based axoneme, which also allows for bidirectional transport of cargoes into and out of the cilium. Through several types of receptors that cross its membrane, the PC can transduce multiple kinds of chemical and mechanical stimuli within the cells [20,21,22,23]. PC is present in the majority of tendon cells, and is oriented to the direction of collagen fibers [24]. In tendons, changes in cilia properties in response to loading and matrix environment suggest a role in mechanotransduction [25]. Several intracellular molecular pathways including those related to Hedgehog (Hh), Wnt, Notch, and Hippo, as well as downstream of G-protein coupled receptors (GPCRs), platelet-derived growth factor receptors (PDGFRs), and other receptor tyrosine kinases (RTKs) including fibroblast growth factor (FGF) and transforming growth factor beta receptor (TGF-βR), are related to cilia activity [21,22]. Among them, the Hh pathway, which has a role in cellular proliferation and differentiation, seems to be primarily related to the PC [26].

Integrins are transmembrane receptors that bind cells to the ECM and sense changes in the elastic properties of ECM components. In vitro studies allowed us to identify several integrins capable of binding to collagen VI [1]. Activated integrins engage several intracytoplasmic proteins forming focal adhesions (FAs), dynamically clustered molecular platforms containing actin-binding proteins, such as talin, which mediate actin remodeling. The actin cytoskeleton mechanically transmits mechanical cues from cell surface receptors to the cytoplasm and nucleus, thereby regulating cell shape by tensioning the cell to the matrix [27]. FAs also activate several protein kinases including FA kinase (FAK), leading in turn to the activation of Akt and extracellular signal-regulated kinase ½ (ERK1/2), which phosphorylate specific substrates and amplify the signal inside the cells. Downstream of FAs, key transcriptional regulators, such as Yes associated protein-1 (YAP) [28], regulate the expression of mechanoresponsive genes, also playing a crucial role in the growth and regeneration of cells and organs [29,30].

In this study, we tested the hypothesis that PCM defects due to collagen VI deficiency may affect the response of cultured TFs to mechanical stimulation. By generating cell cultures from tendons of healthy human subjects and of patients affected by UCMD, we found that the cellular response of UCMD TFs to mechanical stimulation is impaired at multiple levels. Our data reveal that PC structure and Hh signaling are not properly recovered in cultured UCMD tenocytes after mechanical stimulation. This is paralleled by defective regulation of FAs, talin and actin remodeling, as well as of the molecular pathways headed by Akt, ERK1/2, and p38MAPK, and of the activity of mechanoresponsive genes downstream of YAP.

## 2. Materials and Methods

### 2.1. Patients

Tendon biopsies of four unaffected donors and three genetically characterized UCMD patients [7] were used for the study. Biopsies were isolated from the pedidium tendons of two UCMD patients carrying a heterozygous c.896G>A mutation in *COL6A1* (p.Gly299Glu, UCMD1), and a homozygous c.2572C>T mutation in *COL6A2* (p.Gln858X, UCMD2), respectively, and from the Achilles tendon of one UCMD patient with a heterozygous c.850G>A mutation in *COL6A1* (p.Gly284Arg, UCMD3). Samples from control tendons were harvested during elective surgery involving the lower extremity in age-matched volunteer controls. In particular, the two pedidium and the two Achilles tendons were harvested from patients operated on for the internal fixation of fractures at the tibial plafond (*n* = 2) and for surgically neglected clubfoot correction by means of osteotomy (*n* = 2), in which the respective tendons were exposed during the surgical approach. In all these patients, the harvested tendons were not involved in any known pathological process. All subjects gave their informed consent before participating in the study. The study was conducted in accordance with the Declaration of Helsinki, and the protocol was approved by the Ethics Committee at the Rizzoli Orthopedic Institute (project identification code: CE 0007151, 11 May 2021).

### 2.2. Tendon Cell Cultures

Tenocytes, the mature cell type of the tendon, have a low proliferative capacity, thus we established tendon cell cultures mainly derived from progenitor cells, which display a fibroblast-like phenotype [8]. In the text, we refer to tendon-derived cultures as TFs. To obtain TFs, tendon fragments were subjected to mechanical dissociation, and the cells were grown in Dulbecco’s modified Eagle medium (DMEM) supplemented with 1% antibiotics and 10% fetal bovine serum (FBS) [10]. Cells were maintained in a humidified atmosphere with 5% CO_2_ at 37 °C, grown to confluence, and treated with 0.25 mM L-ascorbic acid for the time indicated, to promote collagen VI secretion [31]. Considering the well-documented effect of ascorbic acid on cell proliferation and mechanosignaling-related pathways [32,33], ascorbate treatment was suspended during loading experiments. All in vitro experiments were performed on cells at three to seven passages from the initial isolation. Before each experiment, cellular senescence was evaluated by subjecting healthy cultures to a beta-galactosidase assay, and to Ki-67 and MitoTracker deep red staining.

### 2.3. Immunofluorescence and Confocal Microscopy

Immunofluorescence analysis with anti-collagen VI antibody (Millipore, Temecula, CA, USA) was performed on TFs, as previously reported [9]. Cells grown on coverslips were incubated with antibodies against collagen I (Abcam, Cambridge, UK) and collagen XII (Santa Cruz Biotechnology Inc., Santa Cruz, CA, USA). Focal adhesions were stained with Alexa fluor 568-conjugated phalloidin (1:200, Invitrogen, Thermo-Fisher Scientific, Waltham, MA, USA), antibodies against anti-phosphorylated Y397 Focal Adhesion Kinase (pFAK), and talin (Merck-Sigma, St. Louis, MO, USA). The PC axoneme was labeled with antibodies against acetylated α-tubulin (Merck-Sigma) and ADP ribosylation factor like GTPase 13b (Arl13b, Proteintech, DBA Italy, Segrate, Italy). Primary antibodies were revealed with secondary antibodies conjugated with fluorescein isothiocyanate (FITC), tetramethylrhodamine (TRITC), and Cy5 (DAKO, Agilent, Santa Clara, CA, USA). Cell nuclei were stained with 4′,6-diamidino-2-phenylindole (DAPI; Merck-Sigma). Samples were mounted with an anti-fading reagent (Invitrogen) and observed with a Nikon Eclipse epifluorescence microscope. Confocal imaging was performed with a Nikon A1-R confocal laser scanning microscope as previously described [10]. To process 3D images, 20–25 consecutive confocal images were stacked up with surface shaded reconstruction. No deconvolution was applied to the images. Working dilutions of the antibodies used are provided in Supplementary Table S1.

### 2.4. Mechanical Strain

TFs were cultured onto silicon flexible-bottomed culture plates coated with collagen I (BF-3001C-BioFlex-Collagen Type I, Dunn Labortechnik GmbH, Asbach, Germany) for uniaxial stretch in DMEM with 10% FBS, 1% antibiotics, and anti-mycotic. The FlexCell Tension Plus system FX-4000 (Flexcell International Corporation, Hillsborough, NC, USA) was used to apply a tensile strain. Uniaxial, cyclic mechanical tension was applied to cells with physiological (4%) elongation and a frequency of 0.5 Hz for 22 h, in a humidified atmosphere with 5% CO_2_ at 37 °C as described [25]. For recovery experiments, at the end of the stimulus, cells were maintained in unstretched conditions for the indicated times. Stretched cell cultures were then subjected to immunofluorescence, western blot, RT-qPCR, and scanning electron microscopy analysis.

### 2.5. Scanning Electron Microscopy

Cells were fixed with 2.5% glutaraldehyde and 0.1 M cacodylate buffer, pH 7.3, for 1 h at 4° C. After washing with 0.1 M cacodylate buffer, cells were post-fixed with 1% osmium tetroxide in 0.1 M cacodylate buffer for 1 h at room temperature. After rinsing with 0.1 M cacodylate buffer, samples were dehydrated in an ethanol series, critical point dried, sputter-coated with gold, and observed at 0° tilt angle with a Zeiss Evo MA10 scanning electron microscope. Acceleration voltage was set at 30 kV and WD at 15 mm.

### 2.6. Transmission Electron Microscopy Studies

Tendon biopsies were fixed with 2.5% glutaraldehyde and 1% osmium tetroxide in 0.1 M cacodylate buffer and embedded in Epon812 epoxy resin following standard procedures. Ultrathin sections were stained with uranyl acetate and lead citrate. For rotary shadowing, tendon fibroblasts were grown on coverslips and, after confluence, treated for 24 h with 0.25 mM L-ascorbic acid. In vitro immunolabeling was performed with a mouse monoclonal antibody against collagen VI α3 chain globular domain (Millipore, Burlington, MA, USA). Rotary shadowing of immuno-gold labeled samples was performed following previously established procedures [9]. Replicas were washed with distilled water and collected on copper grids. Both Epon812 embedded ultrathin sections and replicas were observed with a JEOL JEM 1011 transmission electron microscope operated at 100 kV.

### 2.7. Protein Extracts and Immunoblot

Strained, recovered, and unstimulated cells were washed in PBS and scraped in SDS-lysis buffer [34]. A total of 10–20 µg of lysates were resolved on 4–15% precast polyacrylamide gel (Bio-Rad Laboratories SrL Italy, Segrate, Italy), transferred onto nitrocellulose membranes (Santa Cruz Biotechnology, DBA SrL Italy, Segrate, Italy), and immunoblotted with the antibodies listed in Supplementary Table S1. Chemiluminescence images were taken using the ChemiDoc MP Imaging System (Bio-Rad, Hercules, CA, USA).

### 2.8. RNA Extraction and Gene Expression Analysis

Total RNA was extracted from 0.2 × 10^6^ cell samples using 0.5 mL TRIzol Reagent (Invitrogen), according to the manufacturer’s instructions, and quantified with Nanodrop ND-1000 (Nanodrop Technologies, Wilmington, DE, USA). Reverse transcription was performed with 600 ng total RNA and M-MLV Reverse Transcriptase (Thermo-Fisher Scientific), using random hexamers. The resulting cDNAs were processed for quantitative real-time PCR using Hot Firepol Eva Green qPCR mastermix (Solis BioDyne) and a QuantStudio TM 5 Real-Time PCR system (Thermo-Fisher Scientific). Each sample was loaded in triplicate and analyzed through the Design and Analysis 2.6.0 software (Thermo-Fisher Scientific). *RPLP0*, coding for ribosomal protein lateral stalk subunit P0, was used as a housekeeping, or control, gene. Primer sequences are provided in Supplementary Table S2.

### 2.9. Image Processing and Statistical Analysis

Morphological and biochemical images were processed by Photoshop CS4 (Adobe Systems, Adobe, San Jose, CA, USA). Artwork was created by Microsoft PowerPoint. Densitometric analyses were performed by ImageJ (National Institute of Health). Except where indicated, data are expressed as the mean ± SD of the number of the reported biological replicates. Statistical significance was measured by one-way ANOVA with Tukey’s multiple comparison test (for more than two comparisons) and Student’s *t* test (comparison of two groups). Statistical analyses were carried out using GraphPad Prism version 5.0 for Windows (GraphPad Software, La Jolla, CA, USA). The results were considered statistically significant for *p* values less than 0.05.

## 3. Results

### 3.1. Mutations in COL6A1 and COL6A2 Genes Affect the Organization of Collagen VI in the ECM of TF Cultures, and the Structure of Collagen Fibrils in the Mature ECM of Tendons

To characterize the effect of collagen VI mutations on the protein organization and distribution in TF cultures, immunofluorescence analysis with anti-collagen VI was performed on cells grown to confluence. In particular, we investigated cell cultures derived from tendon fragments of two UCMD patients carrying mutations in the COL6A1 gene (UCMD1 and UCMD3), and one in the COL6A2 gene (UCMD2).

**Figure 1:**
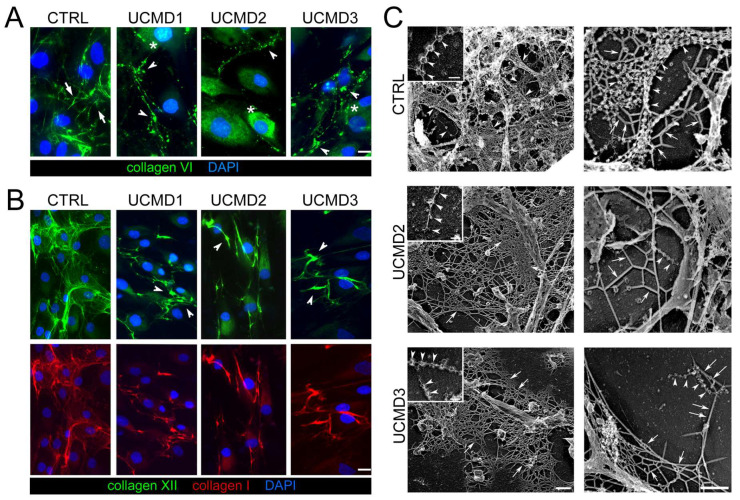
Immunofluorescence and ultrastructural changes of collagen VI in UCMD tendon cultures. (**A**) Representative immunofluorescence for collagen VI (green) in TF cultures from control (CTRL) and UCMD (UCMD1-3) patients. Cells were grown to confluence and maintained 4 days in the presence of 0.25 mM L-ascorbic acid. In control cultures, collagen VI formed a dense extracellular network constituted by interconnected microfibrils (arrows). UCMD cultures displayed an altered arrangement of collagen VI networks, featuring dot-like deposits (arrowheads) in the ECM. In UCMD2 and UCMD3, the protein also accumulated in the cytoplasm (asterisks) of cells. (**B**) Representative image for double labeling with anti-collagen XII (upper panels, green) and collagen I (lower panels, red) in TF cultures, showing a coarse ECM arrangement (arrowheads) in UCMD cultures. In A and B, nuclei were stained with DAPI. Scale bar, 10 μm. (**C**) Transmission electron microscopy visualization of rotary shadowed replicas of proliferating TFs from control (CTRL), UCMD2, and UCMD3 patients, immunolabeled with anti-collagen VI and 5 nm-colloidal gold conjugated secondary antibody. In normal controls, colloidal gold particles associate with the globular domain of collagen VI tetramers, that, in turn, form microfilaments featuring “pearl necklace strands” (left upper panel, inset, white arrowheads), and complex webs. Other components of the ECM (upper right panel, white arrows), appear almost completely masked by the complex collagen VI webs. Note that in both UCMD TFs’ cultures, the few secreted collagen VI tetramers show irregular globular domains (left panels, white arrowheads): they form short microfilaments (right panels, white arrowheads), and the ECM, in general, due to the absence of collagen VI, has a smooth appearance (white arrows). Scale bar, 0.1 μm.

Results revealed that in control cells, collagen VI was mainly in the ECM, with the typical filamentous arrangement, forming a well-developed network of interconnected microfilaments (Figure 1A). However, in UCMD cultures, collagen VI was prevalently retained in the cytoplasm of cells, with a diffuse non-filamentous pattern, (Figure 1A), and minimally in the ECM, where it was assembled in few filaments and aggregates with a spot-like appearance (Figure 1A). In addition, collagen I and collagen XII, two main components of the tendon ECM also known for functionally interacting with collagen VI [35,36], also displayed an altered pattern in UCMD cultures, forming anomalous aggregates (Figure 1B).

The organization of collagen VI secreted in the ECM of TFs cultures was further explored by electron microscopy analysis of rotary shadowed replicas of cultures. This experimental approach generally allows a fine definition of the organization of the ECM network at the early stage of its assembly. Collagen VI microfilaments were immunolabeled with an anti-collagen VI specific antibody. In control replicas, typical webs of collagen VI microfilaments appeared abundantly expressed and well interconnected with the cell processes and the ECM network, a pattern consistent with the proposed function of collagen VI in mediating the attachment of cells to the ECM. In contrast, UCMD TF cultures displayed few and short single microfilaments or amorphous protein aggregates rarely associated with the cell processes and ECM network (Figure 1C).

To get insights into the impact of collagen VI mutations on the structure of mature ECM, tendon biopsies from UCMD2 and UCMD3 patients were analyzed by transmission electron microscopy and the results were compared to age-matched control biopsies. A normal tendon matrix appeared mainly represented by collagen fibrils of different diameters, and the analysis of diameters revealed a bimodal distribution, with most of the fibrils ranging between 20 and 170 nm (Supplementary Figure S1). The analysis of UCMD tendon biopsies showed alterations of the collagen fibrils’ morphology, which indeed appeared fragmented with irregular profiles, while the distribution of collagen fibril diameter was shifted toward smaller diameters (50–120 nm in both UCMD tendons), with a loss of fibrils having a diameter >140 nm in the pedidium tendon of UCMD2 (Supplementary Figure S1B).

These data confirm previous results on tendon biopsies and tendon-derived fibroblast cultures from UCMD1 patients [9,10], and demonstrate that these UCMD mutations affect the ability of collagen VI chains to be assembled in the ECM.

To assess whether collagen VI defects might impair the mechanoresponse of tendon cells, we next focused on two major mechanosensitive cellular structures and their associated molecular machinery, namely PC and FAs.

### 3.2. Mechanical Stress Impairs PC Morphological and Functional Properties in UCMD TFs

**Figure 2:**
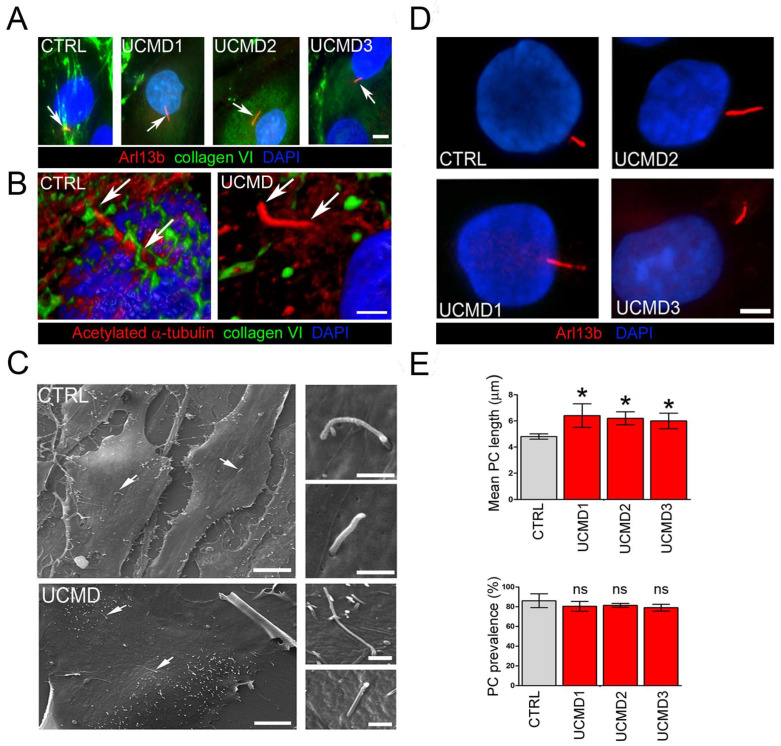
PC organization and structure in control and UCMD tendon cultures. (**A**) Representative double immunofluorescence for Arl13b (red) and collagen VI (green) in control (CTRL) and UCMD (UCMD1-3) TFs. Arrows indicate the PC. Note the absence of collagen VI microfibrils in the proximity of the PC in all three UCMD cultures, opposite from the dense collagen VI network enveloping the PC in control cells. Nuclei were stained with DAPI. Scale bar, 5 μm. (**B**) Confocal imaging and 3D reconstruction of a single PC in control (CTRL) and collagen VI-deficient (UCMD) TFs, double-labeled with antibodies against collagen VI (green) and against acetylated α-tubulin (red), confirming the absence of collagen VI associated with the surface of the PC in UCMD TFs. Arrows indicate the extension of the cilium axoneme. Scale bar, 2 μm. (**C**) Scanning electron microscopy of normal (CTRL) and UCMD TFs, showing the presence of the PC (arrows) protruding from the cell surface. Higher magnifications on the right show the typical antenna-like structure of the PC. Scale bar of panels on the left 30 μm, and panels on the right 2 μm. (**D**) Representative immunofluorescence images of the PC in control (CTRL) and UCMD (UCMD1-3) TFs with an antibody against Arl13b (red), showing longer cilia in UCMD TFs. Nuclei were stained with DAPI. Scale bar, 2 μm. (**E**) Quantification of the PC length (upper panel) and PC prevalence (i.e., number of ciliated cells in percentage; lower panel) in cultured TFs from control (CTRL) and UCMD (UCMD1-3) patients, determined by immunofluorescence with an anti-Arl13b antibody. Measurements were performed with NIS AR 4.50 software, counting 100 cells for each culture. Significance was calculated by one-way ANOVA with Tukey’s multiple comparison test and was expressed between each UCMD and control TF under the same experimental conditions. * *p* < 0.05; ns, not significant; *n* = 3.

The PC is an extracellular antenna-like organelle that protrudes in the PCM, sensing both chemical and mechanical changes in the extracellular environment. By the activation of several downstream signaling pathways, the PC then triggers the adaptation to stress. To investigate whether the observed collagen VI defects in the PCM of UCMD TFs impacted the structural organization of the PC, TFs from UCMD patients and control donors were double stained with antibodies against collagen VI and components of the PC axoneme, namely Arl13b and acetylated γ-tubulin. The analysis revealed that in control cells, the PC was surrounded by collagen VI microfilaments. On the contrary, in UCMD TFs, the anomalous deposits of the mutated protein were never associated with the PC surface (Figure 2A,B). Scanning electron microscopy did not show any overt change in PC morphology in UCMD TFs when compared to controls, with the exception of cilia length, which appeared increased in UCMD cells (Figure 2C). Interestingly, a morphometrical analysis of PC length and prevalence (i.e., the percentage of cells with PC) showed a significant increase in the PC length in UCMD TFs, but similar values of PC prevalence (Figure 2D,E).

**Figure 3:**
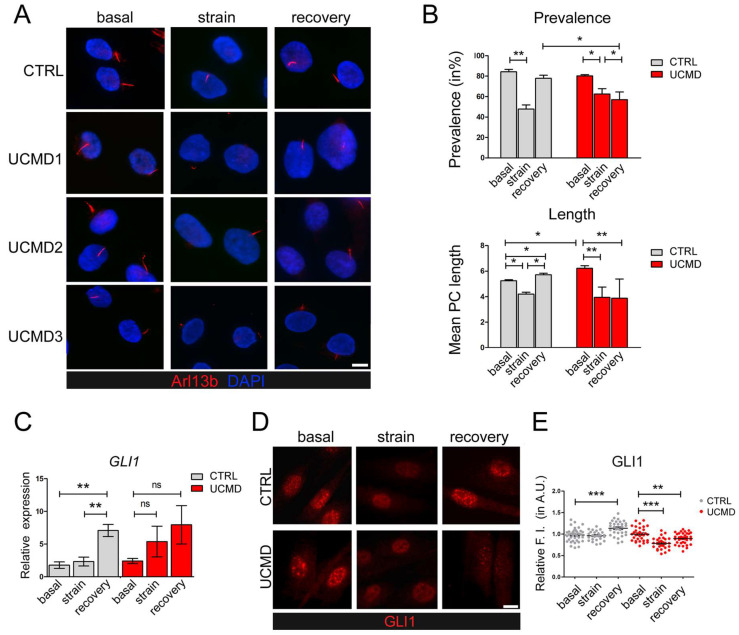
Modulation of PC and Hh signaling in control and UCMD tendon cultures under mechanical stress. (**A**) Representative immunofluorescence analysis of the PC in control (CTRL) and UCMD (UCMD1-3) TF cultures labeled with anti-Arl13b antibody (red), under unstrained condition (basal), after uniaxial strain (strain), and after a 3 h recovery from strain (recovery). Nuclei were stained with DAPI. Scale bar, 5 μm. (**B**) Quantification of PC prevalence (upper panel) and average PC length (lower panel) in control (CTRL) and UCMD TFs under the same conditions as in panel A. Statistical analysis by one-way ANOVA with Tukey’s multiple comparisons test. *, *p* < 0.05; **, *p* < 0.005; *n* = 3. (**C**) RT-qPCR analysis of GLI1 expression in control (CTRL) and UCMD TFs maintained in the above three conditions. RPLP0 served as the internal reference. Data are expressed as 2^−ΔCt^ × 10^5^ and presented as mean ± SD of at least three biological replicates. **, *p* < 0.001; ns, not statistically significant; *n* = 4 control cultures, each condition; *n* = 3 UCMD cultures, each condition. (**D**) Representative immunofluorescence for GLI1 in control (CTRL) and UCMD TFs maintained in the above three conditions. Scale bar, 5 μm. (**E**) Quantification of the nuclear relative fluorescence intensity (F.I.) of GLI1 staining in control (CTRL) and UCMD TFs, based on GLI1 immunofluorescence as in D. For each culture, values were normalized on the respective basal condition. Measurements were performed with NIS AR 4.50 software, counting 70 cells for each culture. Statistical analysis by one-way ANOVA with Tukey’s multiple comparisons test. ** *p* < 0.005, *** *p* < 0.0001; ns, not significant; *n* = 3.

Starting from the evidence of PC changes in UCMD tendon cells, we evaluated whether and how extracellular stimuli might exacerbate such alterations. Towards this aim, cultured UCMD and control TFs were seeded on compliant membranes, grown in the presence of ascorbic acid for four days after confluence, and subjected to uniaxial stretching. To avoid effects on cell cycle [32], which are known to influence ciliogenesis [37], mechanical stimulation was performed in the absence of ascorbate. Following application of mechanical strain, both control and UCMD TFs were properly oriented, with their major axis aligned perpendicularly to the axis of stretch (Supplementary Figure S2), in agreement with the need to withstand stress-induced cellular deformation [38]. Confirming previously reported studies [25], immunofluorescence analysis of control TFs with an anti-Arl13b antibody revealed that mechanical stress induced PC disassembly (Figure 3A,B, strain) followed by a full recovery within 3 h of strain removal (Figure 3A,B, recovery). Strikingly, while UCMD TFs responded to mechanical strain disassembling their PC, they did not recover PC prevalence and length following strain removal (Figure 3A,B, recovery).

To evaluate the impact of PC alterations on Hh signaling, a signaling pathway activated downstream of PC [26], we focused on glioma-associated oncogene family zinc finger 1 (GLI1), a specific effector of this pathway whose increased expression and nuclear localization are hallmarks of active Hh signaling [39]. RT-qPCR analysis of control TFs showed that GLI1 transcript levels remained largely unaffected by stretching, while they increased during recovery (Figure 3C). In contrast, UCMD TFs did not display any significant modulation of GLI1 expression, neither under mechanical stimulation nor during recovery (Figure 3C). Immunofluorescence analysis for the subcellular distribution of GLI1 in control TFs confirmed that GLI1 nuclear translocation remained at a steady level in stretched conditions and was increased under recovery from strain (Figure 3D,E). Strikingly, in UCMD TFs, GLI1 nuclear staining was reduced by stretching, and remained at lower levels after recovery, when compared to its own basal condition (Figure 3D,E). In agreement, after recovery, the number of GLI1 positive nuclei was also significantly decreased in UCMD TFs when compared to control cultures (87% of total cells in UCMD vs. 95.3% in controls, *p* = 0.037).

### 3.3. Mechanical Stimulation Impairs the Organization and the Activation of FAs of UCMD TFs

Inside the cells, mechanical stress is also transmitted through the formation of focal adhesions (FAs), which function by physically linking the ECM to the cytoskeleton and triggering the downstream activation of specific responsive genes, enabling the adaptation to stress [40,41].

**Figure 4:**
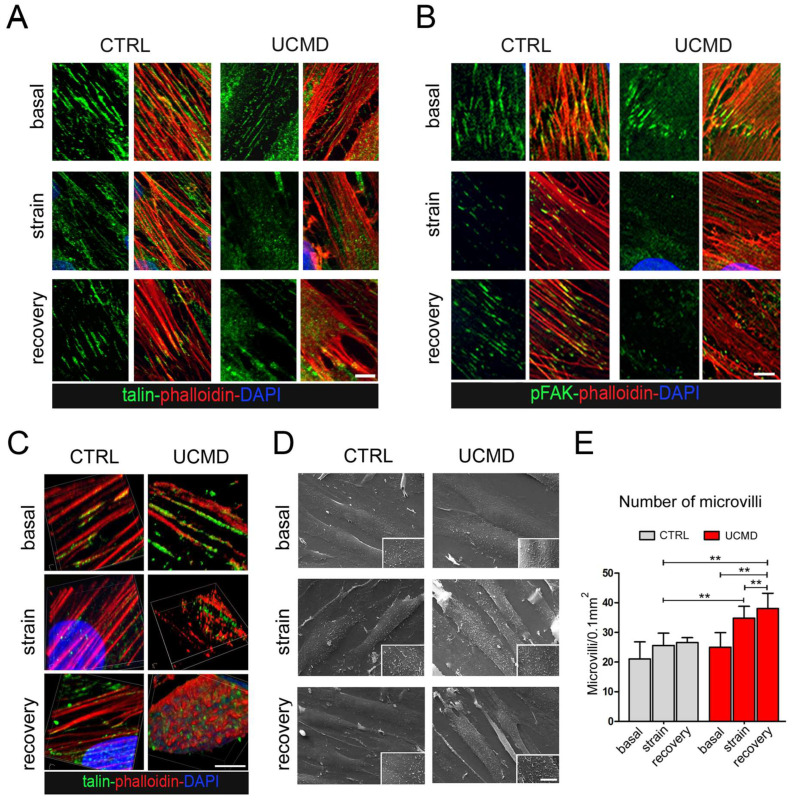
Organization and activation of focal adhesions are impaired in UCMD tendon cultures during and after exposure to mechanical stress. (**A**,**B**) Magnification of confocal imaging of control (CTRL) and UCMD TFs under unstrained conditions (basal), at the end of mechanical stimulation (strain), and upon 3 h recovery from strain (recovery), following double labeling with fluorescent phalloidin (red) and with antibodies against A. talin (green) and B. phosphorylated FAK (pFAK, green). Nuclei were stained with DAPI. Scale bar, 10 µm. (**C**) 3D reconstruction of confocal imaging performed on control (CTRL) and UCMD TFs under unstrained conditions (basal), at the end of mechanical stimulation (strain), and upon 3 h recovery from strain (recovery), labeled with talin (green) and phalloidin (red). Nuclei were stained with DAPI. Scale bar, 5 µm. (**D**) Scanning electron microscopy of control (CTRL) and UCMD TF cultures under unstrained conditions (basal), at the end of mechanical stimulation (strain), and at the end of mechanical stimulation (recovery), showing an increased number of microvilli on the surface of UCMD cells in both stretched and recovery conditions. Scale bar 15 µm for low magnification, 5 µm inset. (**E**) Quantification of the number of microvilli per cell surface area in control and UCMD TFs in the above three conditions, based on scanning electron microscopy imaging as in D. Statistical analysis was performed by Student’s *t* test. **, *p* < 0.05; *n* = 10 fields, each condition.

**Figure 5:**
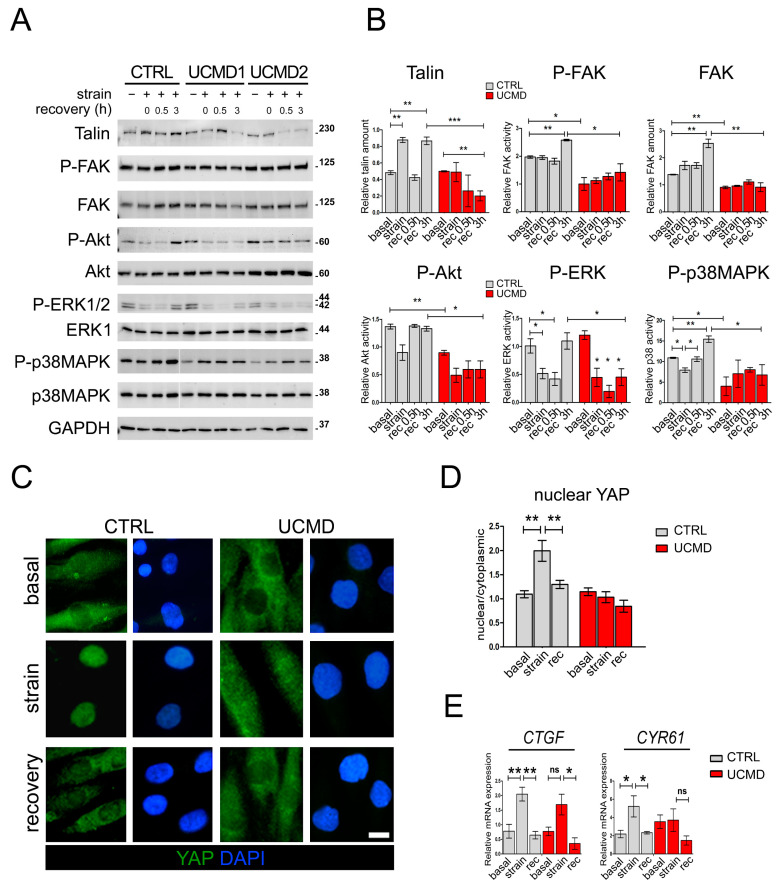
Activation of signaling pathways downstream of focal adhesions is not properly restored in UCMD tendon cultures following recovery from mechanical stress. (**A**) Representative western blot analysis of the expression and the activation profile of talin and different protein kinases in TF cultures of control (CTRL) and UCMD (UCMD1-2) patients in the absence (−) or presence (+) of mechanical strain, as well as upon recovery for the indicated time (0, 0.5 and 3 h). Blots were incubated with antibodies against talin, phospho-Y397 FAK (P-FAK), total FAK, phospho-S473 Akt (P-Akt), total Akt, phospho-T202/Y204 ERK1/2 (P-ERK1/2), ERK2, phospho-T180/Y182 p38MAPK (P-p38MAPK), and total p38MAPK. GAPDH was used as a loading control. Molecular weight markers are reported on the right of the blots and are expressed in kDa. (**B**) Histograms of the relative densitometric quantifications normalized for GAPDH. ***, *p* < 0.001; **, *p* < 0.05; *, *p* < 0.01; *n* = 3, each condition. (**C**) Representative immunofluorescence analysis of the subcellular localization of YAP (green) in control (CTRL) and UCMD TFs under unstrained conditions (basal), at the end of mechanical stimulation (strain), and upon 3 h recovery from strain (recovery). Upon stretching, YAP nuclear staining is displayed by control, but not by UCMD cells. Nuclei were stained with DAPI. Scale bar, 5 µm. (**D**) Quantification of the ratio of the mean fluorescence intensity of nuclear and cytoplasmic YAP, based on immunofluorescence analysis as in C. Data are presented as mean ± SEM. Statistical analysis by one-way ANOVA with Tukey’s multiple comparisons test. **, *p* < 0.002; *n* = 30 measurement, each condition. (**E**) RT-qPCR analysis of CTGF and CYR61 mRNAs levels in control (CTRL) and UCMD TFs under unstrained conditions (basal), at the end of mechanical stimulation (strain), and upon 3 h recovery from strain (rec). Data are expressed as 2^−ΔCt^ × 10^5^ and presented as mean ± SD of at least three biological replicates. **, *p* < 0.005; *, *p* < 0.01; ns, not significant; *n* = 4 control cultures, each condition; *n* = 3 UCMD cultures, each condition.

To evaluate whether the mutated collagen VI of UCMD cells might affect FAs formation, TFs from controls and affected subjects were exposed to mechanical stimulation and analyzed for FA markers and mediators. By double labeling with fluorescent phalloidin, which stains the F-actin, a major component of FAs, confocal imaging of control cultures showed that under basal conditions, talin and active FAK (i.e., phosphorylated at Tyr397) localize along stress fibers. After strain, both talin and active FAK staining were moderately decreased, however, after 3 h of stress removal, the initial pattern was fully recovered (Figure 4A,B, Figures S2B and S3). Under basal conditions, UCMD cultures displayed a pattern of talin and active FAK comparable to that of control cells. However, after mechanical stimulation, both talin and pFAK appeared markedly reduced and disorganized, as evidenced by the loss of colocalization with phalloidin (Figure 4A,B). Since FAs are involved in the remodeling of actin fibers, the organization of actin cytoskeleton was further explored by 3D reconstruction confocal imaging (Figure 4C). The obtained results showed that while in control TFs the cytoskeletal organization of F-actin was minimally affected by mechanical stimulation, in UCMD TFs, F-actin displayed an abnormal/clustered pattern, with linear fibers prevalently developed at the periphery of cells (Figure 4C and Figure S3). These changes were prominent at the perinuclear areas of stretched and recovered UCMD cells (Figure 4C). Scanning electron microscopy showed that the cell surface morphology in UCMD cultures was also deeply rearranged, with an increased number of microvilli in both stretched and recovered conditions, when compared to control cultures (Figure 4D,E). Western blot analysis showed that the failure of talin recovery and of FAK activation displayed by UCMD TFs, corresponded to a reduction in the expression of talin and FAK (phosphorylated and total protein) (Figure 5A).

### 3.4. Akt, ERK1/2 and YAP Activity Are Not Restored in UCMD TFs following Recovery from Mechanical Stress

To investigate whether the defects displayed by UCMD TFs after mechanical stimulation might result in an impairment of specific molecular pathways, we analyzed the activation of major pathways activated by FAs, namely Akt and ERK1/2. Toward this aim, control and UCMD cultures were subjected to mechanical stress, and the phosphorylation of Akt and ERK1/2 kinases assessed after 30 min and 3 h of recovery from the end of the stimulus. In control TFs, after a transitory reduction, the levels of both phospho-Akt and phospho-ERK1/2 were completely restored to those observed under unstimulated conditions (Figure 5A,B). Conversely, in UCMD TFs, the levels of phospho-Akt and phospho-ERK1/2 failed to recover at both time points from the end of the mechanical stimulus (Figure 5A,B and Figure S4). Interestingly, western blot analysis for p38 mitogen-activated protein kinase (p38MAPK) revealed that the activity of this kinase was restored in both control and UCMD TFs following the removal of mechanical stimulation. However, whereas in control cells mechanical loads induced a significant reduction in p38MAPK activity, in UCMD samples it did the opposite (Figure 5A,B). Together, these data pointed at an altered reception and transduction of the mechanical signal in UCMD tendon cells.

Upon mechanical stimulation, in control cultures YAP was displaced in the nuclei of the cells. However, in UCMD counterparts, YAP labeling was mainly found in the cytoplasm but faintly in the nuclei (Figure 5C,D). YAP nuclear retention of control TFs was highly dynamic, as after 3 h of recovery from stress, the majority of this protein relocated to the cytoplasm, with a small amount still located in the nucleus (Figure 5C,D). Of note, in UCMD TFs, the distribution of YAP staining remained largely unchanged even after 3 h of recovery from stress (Figure 5C,D). To confirm that nuclear translocation of YAP resulted in the activation of specific responsive genes, we analyzed the expression of two well-characterized YAP target genes, namely connective tissue growth factor/cellular communication network factor 2 (CTGF/CCN2) and cysteine-rich angiogenic inducer 61/cellular communication network factor 2 (CYR61/CCN1). RT-qPCR analysis revealed that mechanical stimulation significantly triggered the upregulation of both CYR61 and CTGF in control cells, whereas UCMD cells were unable to induce a similar increase, most likely because of the lack of an adequate YAP activation (Figure 5E). After 3 h of recovery, the expression of both YAP effectors was completely rescued in control, but not in UCMD cells (Figure 5E). These results showed that the alterations of FAs and FA-related signaling displayed by UCMD TFs upon mechanical stress led to a defective response of YAP and mechanoresponsive genes to strain.

## 4. Discussion

**Figure 6:**
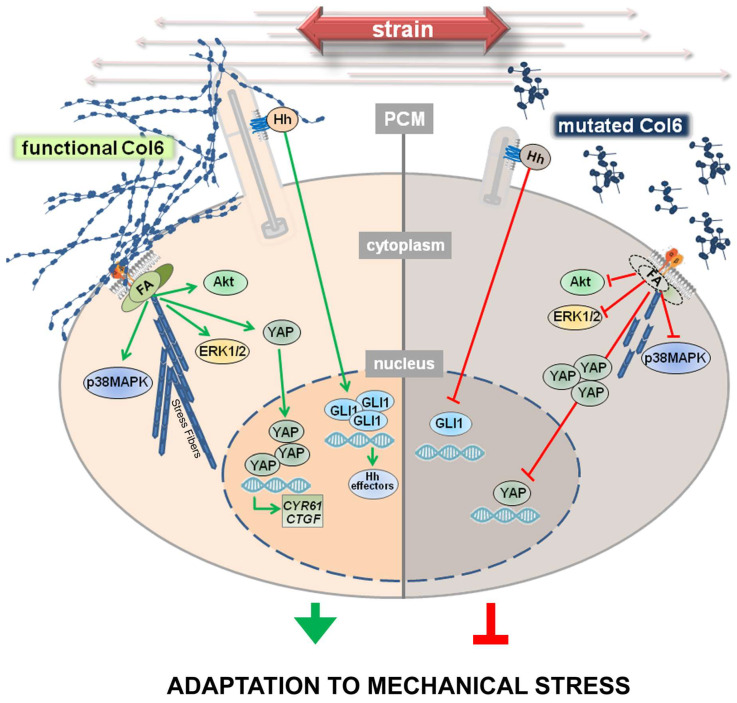
Mutations of collagen VI genes result in defective adaptation to mechanical strain. In normal TFs, following exposure to mechanical strain, at the PCM, the primary cilium (PC) and specific integrins interact with collagen VI, triggering the proper formation of focal adhesions (FA) and stress fibers, as well as the activation of a plethora of molecular pathways that lead to the translocation and activation of GLI1 and YAP. As a result, the upregulation of *GLI1* itself, *CYR61*, and *CTGF* is observed, with possible consequences on ECM remodeling and on the proper cellular adaptation to strain. The absence of a functional collagen VI, here observed on the right, impairs these mechanisms starting with an altered or even absent interaction between collagen VI and the PC, or with specific membrane receptors that result in a poor activation of the downstream molecular pathways. As a consequence, mechanoresponse and mechanotransduction are impaired, possibly leading to an impaired adaptation to stress. Green and red lines indicate active and impaired pathways, respectively.

Although tendon cells live merged in a mechanically stimulated environment, most of the studies reported so far on collagen VI deficient cells have been performed under static conditions. To identify novel molecular pathways underlying pathological mechanisms and their mechanistic links with mutated collagen VI, our study was the first to explore the role of collagen VI in the response to mechanical stimulation of tendon cells, highlighting, for the first time, a novel role for collagen VI as a critical component of the mechanical response of tendon fibroblasts (Figure 6).

Characterization of the ECM of UCMD TFs under basal conditions showed a marked impairment not only in the deposition and organization of collagen VI microfibrils, but also of collagen I and collagen XII, which in COL6-RM TFs formed anomalous aggregates. Of note, these alterations were concomitant to significant changes of the collagen fibrils in tendon biopsies. Starting from these observations, and from our previously reported finding on the failure of affected TFs to correctly polarize upon induction of migration [10], we speculated that collagen VI might play a role in the transmission of mechanical stimulation and hence participate in the mechanoresponse of TFs.

A major role in mechanosensing is played by the PC, an antenna-like sensor that protrudes from the cell membrane in the PCM, interacting with several extracellular components. To understand whether this deficiency might affect PC properties and functions, we investigated PC features in UCMD TFs during the application of mechanical load and following its recovery. Our data demonstrate that cilia of UCMD tendon cells are longer than those of control counterparts and fail to recover their length and prevalence after stretching. Interestingly, during recovery from mechanical stress, UCMD TFs also display impairment of Hh signaling, as revealed by the altered expression and impaired nuclear translocation of GLI1. Although this pathway is generally considered to be specific to the PC, a PC-independent Hh activation was also described [42]. While future studies employing PC-specific inhibitors and tendon cultures from further patients with genetically defined mutations of *COL6A1-A3* genes will provide a thorough dissection of the pathomolecular mechanisms involving cilia and Hh signaling defects in COL6-RM, our findings reveal, for the first time, an impairment of both PC hallmarks and Hh signaling in UCMD TFs during the exposure to mechanical stress. In this respect, it is interesting to consider that literature studies on murine tendons have pointed at Hh signaling as potentially contributing to healing processes, cell cycle, and differentiation [43], as well as to tendon enthesis formation and adaptation to loads [44]. Therefore, alterations of Hh signaling might result in an impairment of tendon integrity during and following injury, or during the maturation of the tissue, or simply upon mechanical activity, with negative consequences on the functions of the entire muscle-tendon unit. Strikingly, as suggested by published evidence [45], functional alterations of the PC might also be responsible for the impaired polarity and migration rate of COL6-RM TFs previously reported by us [10]. Several pathways involved in ciliogenesis and cilium elongation have already been described. Among these, many are altered in UCMD conditions and concern, for example, autophagy [46], whose defect is a hallmark of COL6-RM [1,11], or remodeling of cortical actin [47], which is altered in our model. Strengthening the potential relevance of the PC in COL6-RM, substantial evidence is provided by a group of disorders featuring mutations of genes encoding for ciliary proteins, called ciliopathies. Some of these conditions display tissue and subcellular alterations similar to those found in COL6-RMs, and include, for example, deregulated cell growth and polarity, dysfunctional autophagy [46], defects of ECM components, enhanced expression of matrix metalloproteinases and TGF-β, and extensive fibrosis (for a comprehensive review, see [23]). The last evidence suggests the involvement of the PC in the remodeling of ECM, which has been demonstrated in some cellular models [48]. Intriguingly, patients affected by the most severe forms, including for example Meckel-Gruber syndrome [49,50,51,52], also feature joint contractures. In this context, it will be challenging to explore if the tendons and muscles of these patients are morphologically altered, or if changes in the quality and the amount of the single components of the ECM are present.

FAs are dynamic molecular platforms that cluster at the cell membrane following the interaction between integrins and ECM components. Our results show that in UCMD TFs, mechanical stimulation triggers the formation of defective FAs, with abnormal organization of FAK and the actin-binding protein talin. FA defects are paralleled by an altered pattern of F-actin, as well as by a defective modulation of the activity of Akt, ERK1/2, and p38MAPK. Of note, previous studies show that decreased ERK1/2 activity is a trait of diseased tenocytes, responsible for increased susceptibility to IL-1β and resulting in an increase in the fibrotic mediator TGF-β1 [53]. Furthermore, it has been reported that reduced activation of FAK and ERK negatively regulates the differentiation of tendon-derived stem cells [54]. On the other hand, the lack of a proper remodeling of actin in UCMD TFs might have negative consequences on the subcellular redistribution of proteins, such as Akt [55], as well as of organelles, including mitochondria [56] and autophagosomes [57], whose activities are altered in *Col6* null mice [1,11] and in patients affected by COL6-RMs [58]. Finally, it has been reported that COL6 gene expression is activated by AP1 transcription factor [59], which, according to the literature is activated by stress-related signaling pathways headed by p38MAPK and ERK1/2.

Our results also demonstrate that mechanical stimulation of UCMD cells results in an impairment of YAP translocation and activation. Corroborating the impaired molecular signals downstream of YAP, our data indicate that mechanical stress activates the expression of CYR61 and CTGF genes only in control cells. Since CYR61 has been described as an effector of mechanical cues in tendon cells exposed to cyclic-uniaxial stretching, as well as in loaded tendons with large load-bearing capacities, such as Achilles and patellar tendons [60], we can infer that the reduction in YAP activity might have a substantial impact on the physiology of the affected tendons. In the attempt to explore whether there is any relationship between the impaired ciliogenesis and the altered activation of FAs displayed by UCMD cells, we came across some studies indicating that ciliogenesis is promoted by depolymerized actin and inactive YAP [61,62]. Although our present work does not provide experimental evidence for a direct role of dysregulated actin remodeling and/or YAP signaling in abnormal ciliogenesis in UCMD cells, it has been reported that an altered reorganization of F-actin may affect the length of the PC [63], and that cortical actin appears to stabilize existing cilia, while basal body docking and positioning in the cell cortex is also influenced by the distribution of cytoplasmic and apical actin networks [47]. Future studies in this direction will allow us to establish if and how defective FA organization and abnormal PC structure of tendon cells from COL6-RM are interconnected, and whether impaired ciliogenesis is also shared by other cells and tissues with defective matrix deposition of collagen VI. Changes in the cortical actin organization in UCMD TFs are also reflected by the increased number of microvilli at the cell surface of mechanically stressed cells. Microvilli are cell membrane protrusions with an actin-based skeleton, improving the extension of cell surface without affecting cell volume. Microvilli have been detected in normal TFs [64], however, their specific role in tendons has been poorly explored. Considering the involvement of microvilli in several cell functions, including absorption, secretion [65], and mechanotransduction [66], it is possible that in the absence of proper collagen VI deposition, mechanical strain induces an undermined cellular response by acting on different mechanisms and eliciting many altered cell features. The involvement of all the molecular pathways explored in this study, misregulated by mutations of COL6 genes upon mechanical stimulation, has also been associated with molecular mechanisms related to ECM remodeling [67,68,69,70], which generally occurs by synthesis, contraction, and proteolytic degradation of its components [71].

In this scenario, we may speculate that the unproper mechanoresponse observed in UCMD TFs may also impair the normal turnover and remodeling of the PCM upon mechanical stimulation, providing further alterations in resistance to load and force transmission in the tendons of affected patients.

## 5. Conclusions

By exploiting UCMD-derived tendon cells, our study identifies a novel set of morphological and functional alterations that may contribute to the pathogenesis and the onset and progression of the clinical features of COL6-RM, opening up new therapeutic perspectives. Since Hh and YAP pathways are known for being involved in the regulation of proliferation and differentiation of cells, our results also suggest that in UCMD the altered mechanosignaling might have negative consequences on the functions and long-term performance of tendons.

## Data Availability

The datasets obtained during the current study are available upon request.

## Supplementary Materials

The following supporting information can be downloaded at: https://www.mdpi.com/article/10.3390/cells13050378/s1, Figure S1: Ultrastructural changes of tendon biopsies from UCMD patients; Figure S2: Adaptation of tendon cultures to mechanical stress; Figure S3: Focal adhesions of UCMD tendon cultures are not properly recovered after mechanical strain; Figure S4: Full images of western blot data; Table S1: List and concentration of antibodies used in this study; Table S2: List of primers used for RT-PCR experiments.
