# Supplementary material for: Collagen VI Deficiency Impairs Tendon Fibroblasts Mechanoresponse in Ullrich Congenital Muscular Dystrophy
Source: Cells. 2024 Feb 22;13(5):378. doi: 10.3390/cells13050378 (PMC10930931; doi:10.3390/cells13050378)
Supplement: Supplementary file 1 [file cells-13-00378-s001.zip › cells-2805834-supplementary.pdf]

SUPPLEMENTARY FILES

Supplementary Table S1: List and concentration of antibodies used in this study.

Antibody	Dilution	Company
Phospho-Akt (Ser473) #4060	1:1000 (WB)	Cell Signaling
Phospho-p38MAPK (Thr180/Tyr182) #4511	1:1000 (WB)	
p38MAPK #9212	1:1000 (WB)	
ERK1/2 #9102	1:2000 (WB)	
GAPDH #374	1:8000 (WB)	Sigma Merck-Millipore, Darmstadt, Germany
Phospho-FAK (Tyr397) #051140	1:1000 (WB)/1:50 (IF)	
Talin #1676	1:3000 (WB)/1:50 (IF)	
Gli1 #5700740	1:1000 (WB) /1:50 (IF)	
α-tubulin	1:1000 (WB)	
Acetylated α-tubulin	1:5000 (WB)/ 1:500 (IF)	
Phospho-ERK1/2 (pT202/pY204) #SC-136521	1:1000 (WB)	Santa Cruz, Biotechnologies
YAP #SC-376830	1:100 (IF)	
Collagen VI #SC-20649	1:1000 (WB) /1:100 (IF)	
Collagen XII #SC-166020	1:100 (IF)	
Arl13b 66739-1	1:100 (IF)	Proteintech
Secondary antibodies (HRP-conjugated)	1:12000	Abcam Cambridge, UK
Secondary antibodies FITC/TRITC-conjugated	1:200	Abcam Cambridge, UK

Supplementary Table S2. List of primers used for RT-PCR experiments.

GENE NAME	Forward (5’-3’)	Reverse (5'-3’)
hCTGF	AGGAGTGGGTGTGTGACGA	CCAGGCAGTTGGCTCTAATC
hCYR61	CCTTGTGGACAGCCAGTGTA	ACTTGGGCCGGTATTTCTTC
hGLI1	AGGGAGTGCAGCCAATACAG	ATTGGCCGGAGTTGATGTAG

hRPLP0	TGGCAGCATCTACAACCCTG	ACAAGGCCAGGACTCGTTT

Supplementary Figure S1

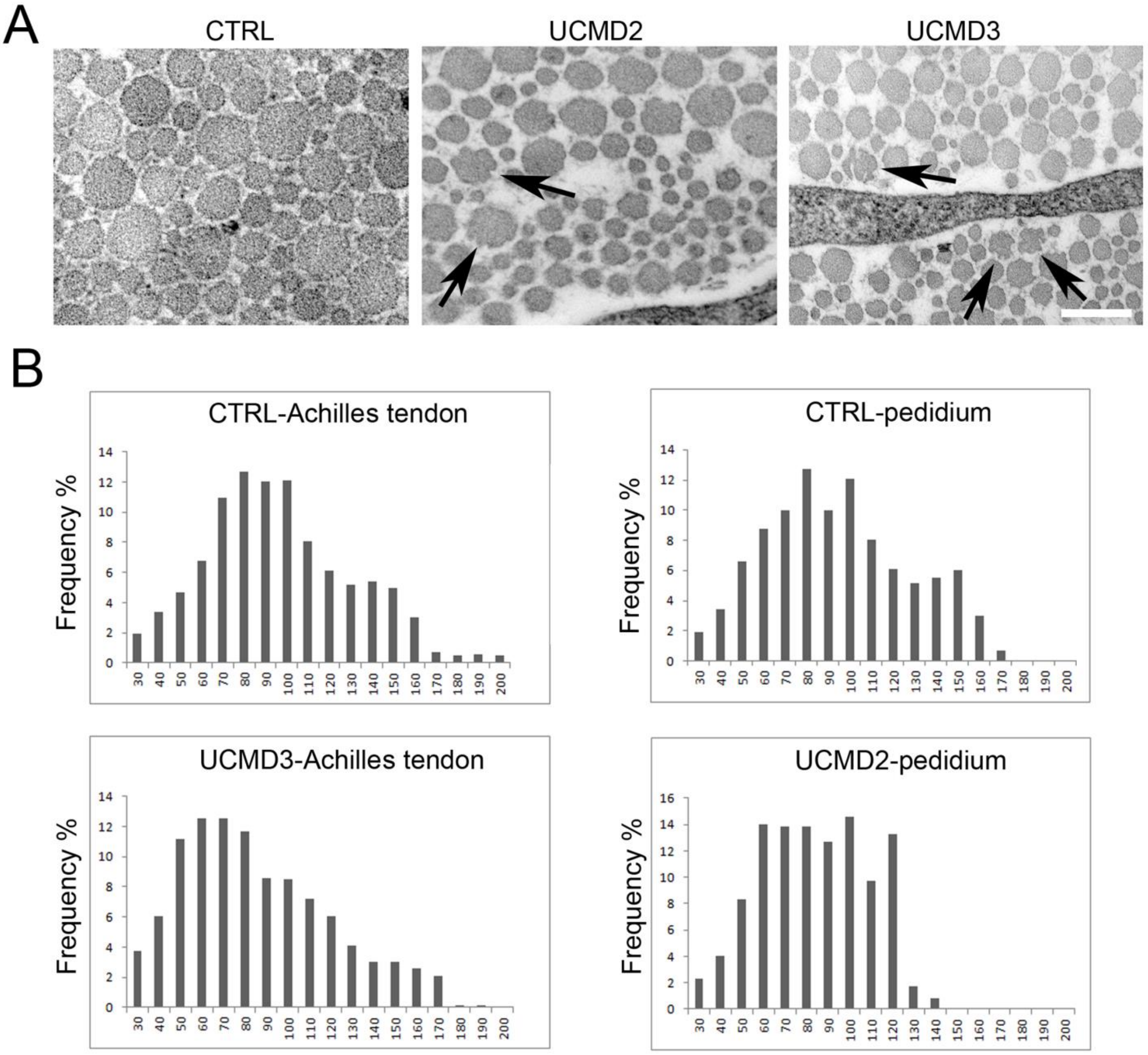

Supplementary Figure S1. **Ultrastructural changes of tendon biopsies from UCMD patients. A.** Representative images of transmission electron microscopy of cross-sectioned normal (control) and UCMD2 and UCMD3 tendon biopsies. Achilles and pedidium tendons from control donors display fibrils with regular profile. In contrast, UCMD tendon displays smaller diameter fibrils; several aberrant fibrils with irregular profile are also observed (arrows). Scale bar, 200 nm. **B.** Fibril diameter distribution in normal and UCMD tendons. The fibrils diameter distribution was shifted toward smaller diameters in UCMD2 and UCMD3 tendon compared to normal control.

Supplementary Figure S2

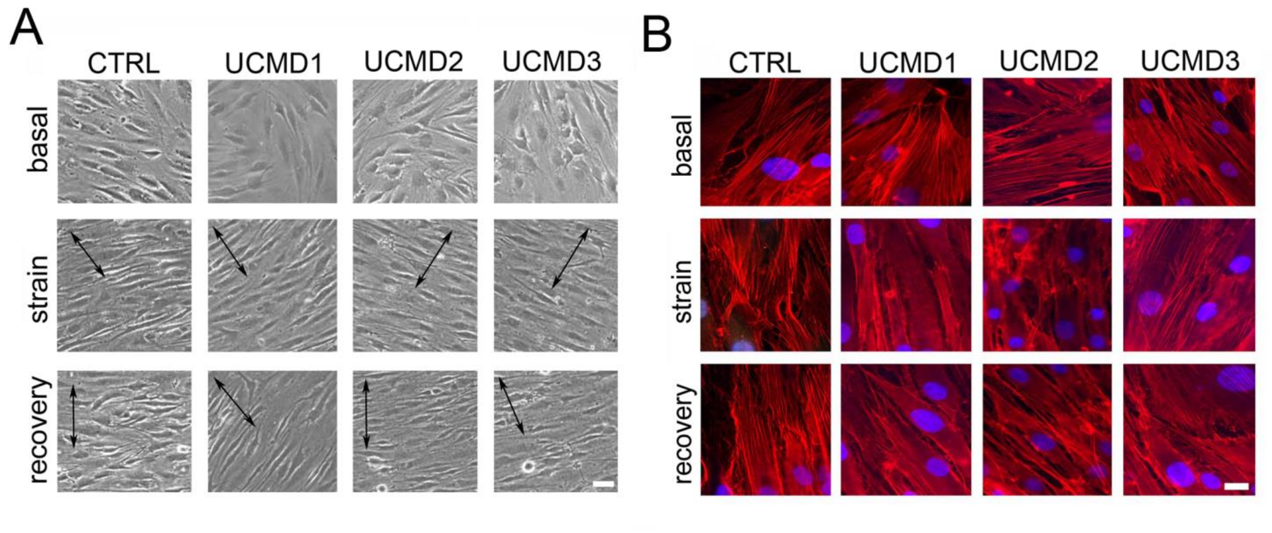

Supplementary Figure S2. Adaptation of tendon cultures to mechanical stress. A. Phase-contrast light microscopy for the morphological evaluation of the spatial alignment of cells in control (CTRL) and UCMD (UCMD1-3) cultures under unstrained condition (basal), following exposure to uniaxial mechanical strain (strain), and upon 3 h recovery from strain (recovery). Arrows indicate the direction of the stretching. Scale bar, 50 μm **B.** Fluorescence microscopy analysis with Alexa-562 conjugated phalloidin in control (CTRL) and UCMD (UCMD1-3) TF cultures maintained in the three above conditions, showing that F-actin linear pattern is lost in UCMD TFs subjected to strain and is not recovered after 3 h of strain removal. Nuclei were stained with DAPI. Scale bar, 5 μm.

Supplementary Figure S3

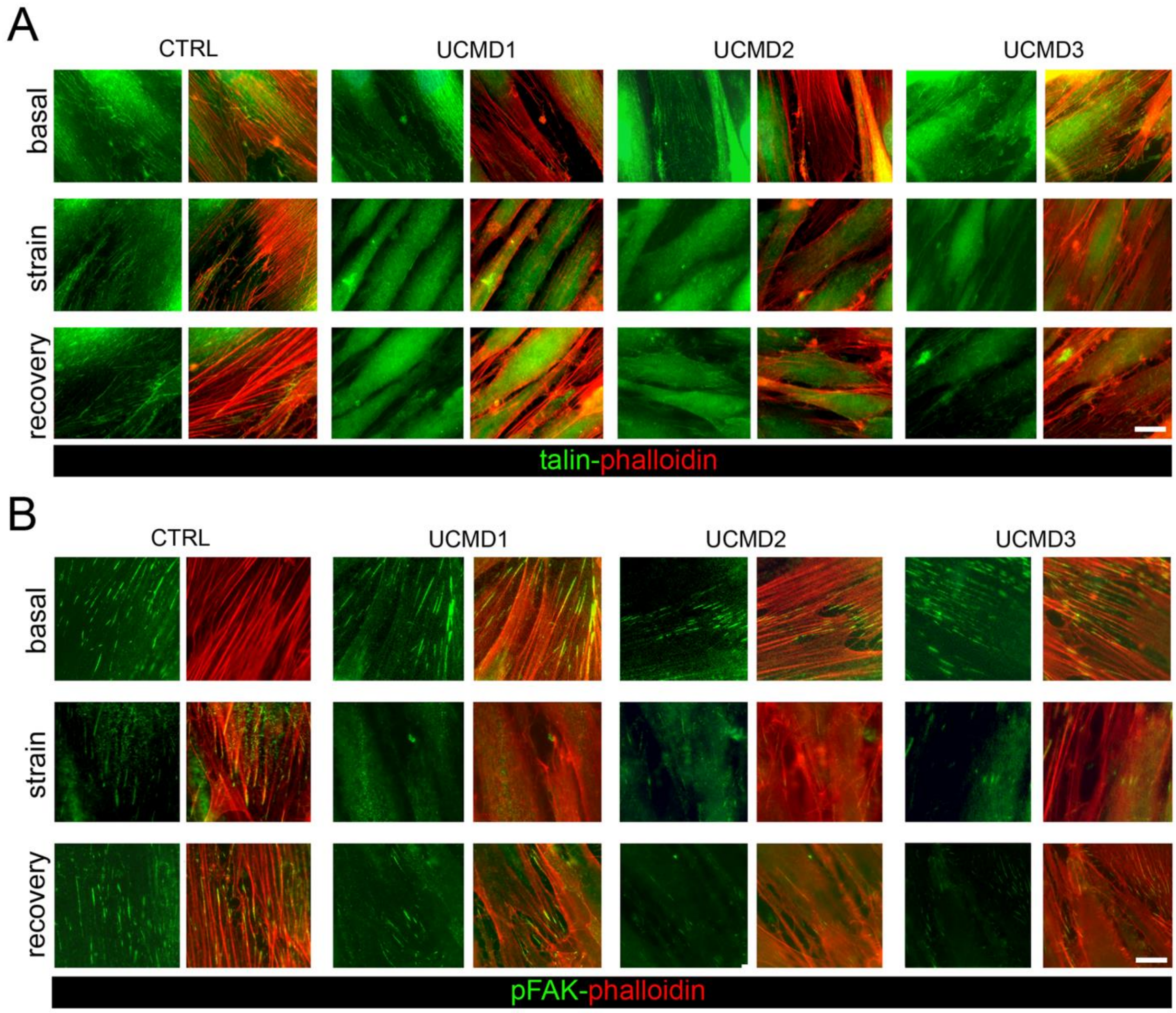

Supplementary Figure S3. Focal adhesions of UCMD tendon cultures are not properly recovered after mechanical strain. A. Immunofluorescence analysis of talin (green) anf F-actin (stained with phalloidin, red) in control (CTRL) and UCMD (UCMD1-3) TF cultures, under unstrained condition (basal), after uniaxial cyclic strain (strain), and after a 3 h recovery from strain (recovery). Scale bar, 5 μm. **B.** Immunofluorescence analysis of pFAK (green) and (phalloidin, red) in control (CTRL) and UCMD (UCMD1-3) TF cultures, under the same conditions as in A. Scale bar, 5 μm.

Supplementary Figure S4

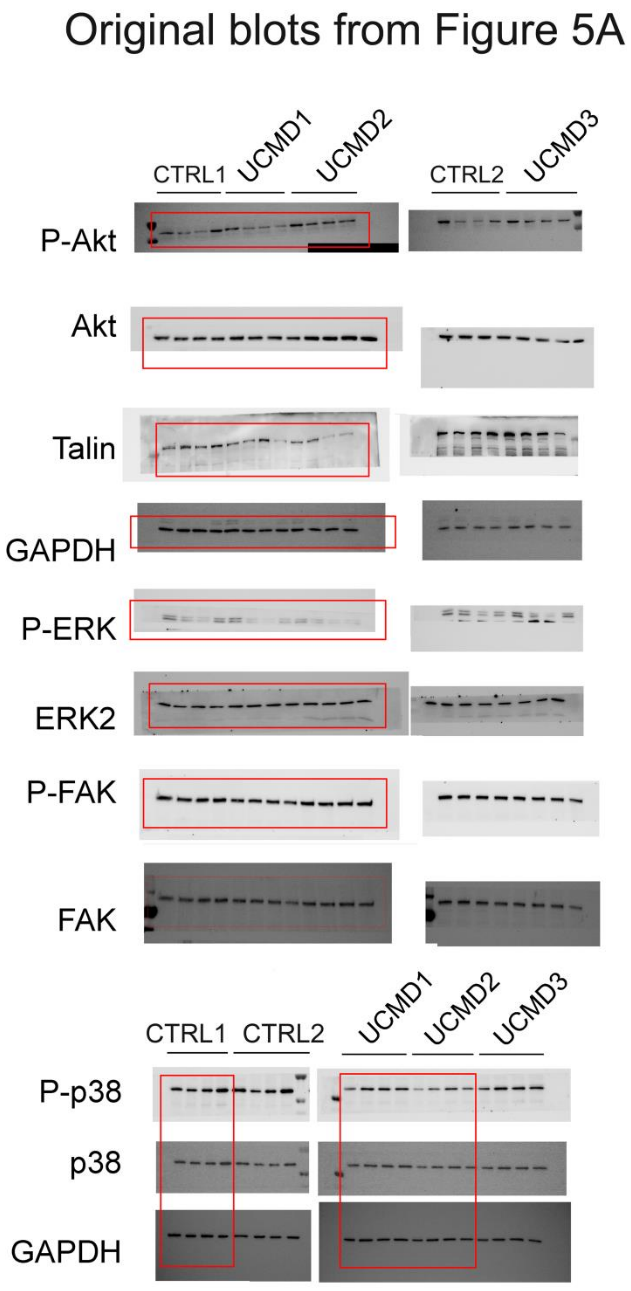

Supplementary Figure S4. Full images of western blot data. The entire gels of western blot experiments shown in Figure 5A are presented, including also CTRL2 and UCMD3. Images were obtained by ChemiDoc™ MP Imaging System (BioRad). Red boxes highlight the portions displayed in the main figures.
